# Percolation analysis of the electrical conductive network in a polymer nanocomposite by nanorod functionalization†

**DOI:** 10.1039/c9ra04680a

**Published:** 2019-11-07

**Authors:** Ruibin Ma, Guangyao Mu, Huan Zhang, Jun Liu, Yangyang Gao, Xiuying Zhao, Liqun Zhang

**Affiliations:** Key Laboratory of Beijing City on Preparation and Processing of Novel Polymer Materials, Beijing University of Chemical Technology 10029 China gaoyy@mail.buct.edu.cn zhaoxy@mail.buct.edu.cn zhanglq@mail.buct.edu.cn; State Key Laboratory of Organic-Inorganic Composites, Beijing University of Chemical Technology 100029 China; Aerospace Research Institute of Materials and Processing Technology Beijing 100076 China

## Abstract

Chemical functionalization of nanofillers is an effective strategy to benefit the formation of the conductive network in the matrix which can enhance the electrical conductivity of polymer nanocomposites (PNCs). In this work, we adopted a coarse-grained molecular dynamics simulation to investigate the effect of the nanorod (NR) functionalization on the conductive probability of PNCs under the quiescent state or under a shear field. It is found that the direct aggregation structure of NRs is gradually broken down with increasing the NR functionalization degree *λ*_A_, which improves their dispersion state. Moreover, a local bridging structure of NRs sandwiched *via* one polymer layer is formed at high *λ*_A_. Corresponding to it, the percolation threshold of PNCs first quickly decreases, then increases and last slightly decreases again with the increase of *λ*_A_, which exhibits an anti N-type under the quiescent state. Meanwhile, it shows a non-monotonic dependence on the interaction between polymer and the functionalized beads which reaches the lowest value at the moderate interaction. However, the percolation threshold is nearly independent of *λ*_A_ under the shear field. Compared with in the quiescent state, the decrease or the increase of the percolation threshold can be tuned by *λ*_A_ under the shear field. The significant change in the percolation threshold is attributed to the orientation and the dispersion state of NRs under the shear field, which affects the conductive network. Especially, we found that the dispersion state of NRs is different for different *λ*_A_ under the shear field. However, the percolation threshold is similar which indicates that the dispersion state of NRs is not completely correlated to the conductive network. In summary, this work presents some further understanding of how the NR functionalization affects the electrical conductivity of PNCs.

## Abstract 2

Chemical functionalization of nanofillers is an effective strategy to benefit the formation of the conductive network in the matrix which can enhance the electrical conductivity of polymer nanocomposites (PNCs).

## Graphical abstract

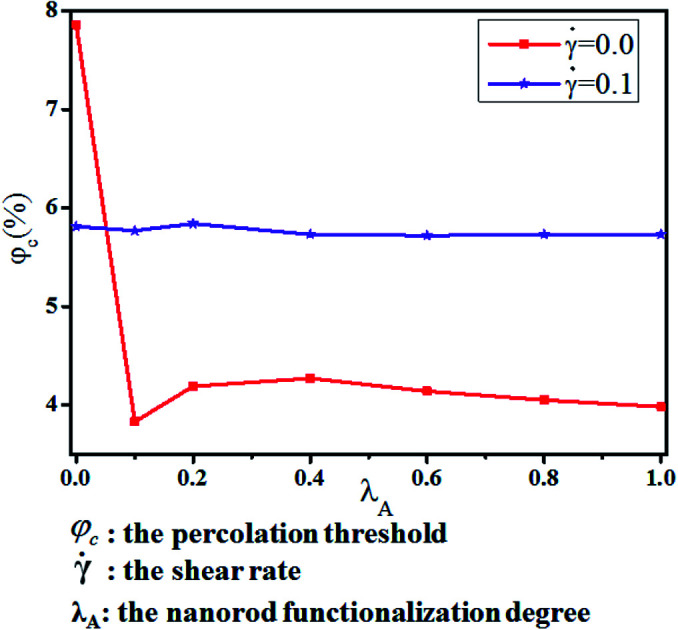

## 1. Introduction

Addition of small amounts of conductive nanofillers (such as carbon nanotubes (CNTs), graphene, carbon black (CB)) into a polymer matrix can increase the electrical conductivity of polymer nanocomposites (PNCs) by orders of magnitude.^1–3^ If the filler concentration is above the percolation threshold, the conductive network will be formed in the matrix which can be understood by percolation theory.^4^ Generally, the formation of the conductive network depends on both the properties of the polymer matrix and the filler and the processing conditions (such as tensile field and shear field).^5–7^ It is important to reduce the percolation threshold of PNCs which can improve the electrical conductivity and reduce cost in industrial applications.

In experiments, a lot of effect is devoted to understand the relationship between the experimental parameters and the electrical conductivity, which aims to enhance electrical property of PNCs.^8,9^ For example, the dependence of the percolation threshold on the aspect ratio of CNTs can be described by a mode which could optimize the CNTs processing conditions.^10^ In addition, the surface functionalization of CNTs is used to form the conductive PNCs with a low percolation threshold.^11,12^ Moreover, the percolation threshold can be further reduced by controlling the CB in one phase of the composite blend.^13^ The tensile and shear fields are necessary during the processing of PNCs which exerts a great influence on the conductive network. Compared with under the quiescent state, the decrease or the increase of the electrical conductivity under the shear field is determined by the initial dispersion state of filler.^14–16^ To better understand how the shear field affects the electrical conductivity, it is necessary to characterize the conductive network.^17,18^ However, experimental techniques are unable to intuitively analyze the variation of the conductive network and electrical properties. Computer simulations have an obvious advantage in analyzing the electrical conductive behavior of PNCs. Currently, by employing a three-dimensional Monte Carlo model, the dependence of the fiber aspect ratio on the percolation threshold is investigated which exhibits an exponential relationship.^19,20^ With the same model, it is found that the aspect ratio of CNTs determines the percolation threshold rather than the tunneling barrier height.^21^ In addition, the size polydisperse will improve the percolation threshold which is approximately inversely upon the weight-averaged aspect ratio.^22^ For comparable sizes of fillers, nanorods can form a percolating network at lower concentration than nanoplates and nanospheres.^23^ Furthermore, it is reported that the average conductive pathway density of CNTs dominates the anisotropy of the electrical property which depends heavily on CNT alignment structure.^24^ By considering the CNT structural distortion in the developed percolation network model, the effect of the temperature on the electrical conductivity is clarified.^25^ The conductive stability of PNCs firstly declines and then rises with the filler concentration at a constant strain, and the minimum corresponds to the percolation threshold.^26^ The shear field will induce the alignment of CNTs parallel to the shear direction which affects the electrical conductivity of PNCs.^27^ Interestingly, as the shear rate is above a critical value, CNTs form the direct contact aggregation which reduces the electrical conductivity.^28^ Especially, the partial alignment of CNTs can result in maximum electrical conductivity of PNCs rather than complete alignment.^29,30^

In fact, because of the self-attraction of CNTs, CNTs show an aggregation structure which is the main cause for the low electrical conductivity of PNCs. Among the various methods,^31–35^ the chemical functionalization of CNTs seems much more effective than others to achieve the uniform dispersion of CNTs in the matrix. However, the effect of the nanorod (NR) functionalization on the conductive behavior of PNCs has not been investigated to our knowledge, especially under the shear field. In this work, by adopting a coarse-grain model, first the microstructure of the NR aggregation is analyzed for different NR functionalization degree. Then, the conductive probabilities of PNCs are calculated under the quiescent state and under the shear field. By calculating the main cluster size and the number of clusters in the conductive network, the relationship between the conductive probability and the NR functionalization degree is well clarified.

## 2. Model and simulation methods

In this work, we adopted a coarse-grained model of the NRs filled PNCs. The classic bead-spring model is adopted to simulate polymer chains which consist of thirty beads.^36^ The total number of polymer beads is fixed to be 42 000 for each system. Each NR contains ten beads, which consists of the strong (A) and weak (B) beads, the only difference being the interaction energy with polymers. The NR functionalization degree is defined as *λ*_A_ = *N*_A_/(*N*_A_ + *N*_B_) which varies from 0.0, 0.1, 0.2, 0.4, 0.6, 0.8 to 1.0 in this work. Here, *N*_A_ and *N*_B_ are the number of A and B beads in one NR, respectively. *σ* and *m* are used to stand for the diameter and mass of each bead respectively. When mapping the coarse-grained model to a real polymer, each bond in this model corresponds to three to six covalent bonds along the backbone of a real chemical chain.^36^

The truncated and shifted Lenard–Jones (TSLJ) potential is adopted to describe the non-bonded interactions between all beads:1
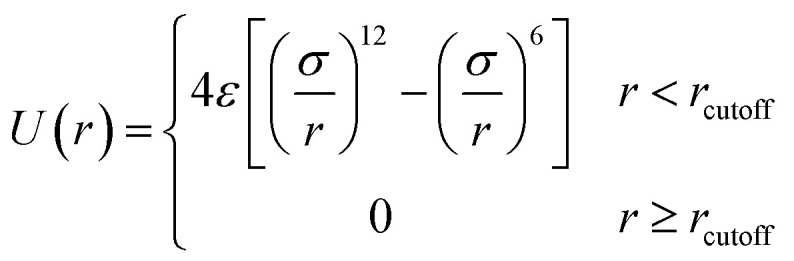
where *r*_cutoff_ stands for the distance at which the interaction is truncated and shifted so that the potential is continuous at *r* = *r*_cutoff_. The polymer–polymer interaction parameter and its cutoff distance are *ε*_pp_ = 1.0 and *r*_pp_ = 2 × 2^1/6^, and the NR–NR interaction parameter and its cutoff distance are set to be *ε*_nn_ = 1.0 and *r*_nn_ = 2.5. The interactions between polymer and A beads on NRs are set to be strong interaction (*ε*_pA_ = 3.0 and *r*_pA_ = 2.5) so that A beads are immiscible with polymer chains. The interaction parameter and its cutoff distance between polymer and B beads are *ε*_pB_ = 1.0 and *r*_pB_ = 2.5, which have a weak attraction with NRs. The random distribution of A monomers in each NR is shown in Fig. S1.† All the related parameters for different interactions are summarized in Table S1.†

The bonded interaction between the adjacent beads including both polymer chains and NRs is represented by a stiff finite extensible nonlinear elastic (FENE) potential:2
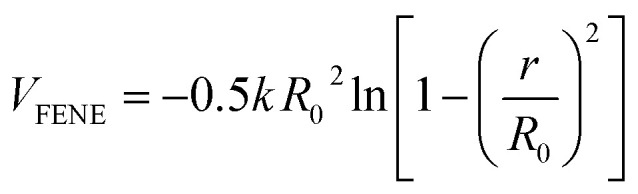
where 
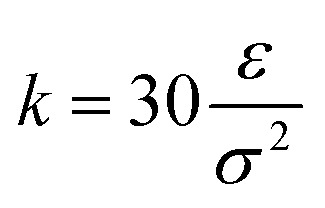
 and *R*_0_ = 1.5*σ*, guaranteeing a certain stiffness of the bonds while avoiding high-frequency modes and chain crossing.

The rod-like character of the NR is enforced by a bending potential, given by

3 *U*_angle_ = *K*(*θ* − *θ*_0_)^2^

where *θ* is the bending angle formed by three consecutive rod beads, *K* is equal to 1000 and *θ*_0_ is set to be 180.

It is noted that it is not our aim to study a specific polymer. Thus, the reduced LJ units *σ* and *ε* are set to be unity, which means that all calculated quantities are dimensionless. Following our previous works,^37–39^ first all the polymer chains and NRs are put into a large box. Then, the NPT ensemble is used to compress the system for 10 000*τ* where *τ* is the reduce time unit. Temperature and pressure are set to be T* = 1.0 and P* = 0.0 respectively by using the Nose–Hoover temperature thermostat and pressure barostat. It is noted that the glass transition temperature of polymer chains is 
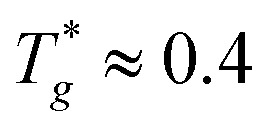
 which is lower than our simulation temperature (T* = 1.0). Periodic boundary conditions are employed in all three directions. The velocity-Verlet algorithm is applied to integrate the equations of motion with a timestep of *δt* = 0.001. Next, a further equilibration is performed for 50000*τ* under NVT ensemble with T* = 1.0. We have checked that polymer chains and NRs have experienced fully relaxed, reaching their equilibrated states for all systems. The equilibrium number density of polymer beads reaches nearly 0.85 which is corresponding to the density of polymer melts. Such equilibrated structures are then used as starting structures for production runs of the structural and dynamical analysis. All simulation runs are carried out by using the large scale atomic/molecular massively parallel simulator (LAMMPS).^40^

It is noted that the electrical properties of the PNCs are correlated to the conductive network. To determine whether the conductive network is formed, a criterion is used to check whether any two NRs are connected. Any two NRs are considered to be connected once their gap is less than the tunneling distance (TD) which can reflect the contact conductance between NRs.^41^ High TD indicates the high contact conductance. Here, the tunneling distance is chosen to be 1.0*σ* from the two aspects (one is the sandwiched polymer chain,^42^ another is that the ratio of CNT diameter to the maximum TD is nearly 1.0 in experiment^43^). It is noted that TD will impact upon the percolation threshold. However, it does not influence the trend of the results. At the beginning of the computational implementation, each NR is assigned a site number and a cluster number. The site number is equal to the cluster number, ranging from 1 to N where N is the total number of NRs. Then, each NR is checked for connection with others. If two NRs are connected, they will be assigned a common cluster number. Finally, all the NRs with the same cluster number are in the same cluster while NRs with different cluster numbers are not connected. Once the NR network spans one direction continuously from one side to another, the system is conductive in this direction. If the NR network spans the three-dimensional directions continuously, it is homogeneously conductive in three directions. Finally, 10 000 equilibrated configurations are dumped which are divided into ten independent blocks. Then we calculated the quantities separately in each block, determine average and standard deviation of the average, and use standard deviation as error bar. The time interval between two continuous frames is 10*τ*. Then, the number of the configurations is counted which is conductive in the three-dimensional directions or in one direction. Here, the conductive probability *Λ*, the directional conductive probability *Λ*_‖_ parallel to the shear field direction, and the directional conductive probability *Λ*_⊥_ perpendicular to the shear direction are used to represent the probability of forming the filler network, which spans the systems in the three-dimensional directions, parallel to the shear direction, and perpendicular to the shear direction respectively. It is noted that the size effect of the simulation box is considered where the conclusions are not changed. Meanwhile, the directional conductive probabilities along the different directions are similar under the quiescent state which indicates that the chosen size is reasonable.

## 3. Results and discussion

### 3.1 Conductive property of PNCs

#### 3.1.1 Effect of the nanorod functionalization degree *λ*_A_

**Fig. 1:**
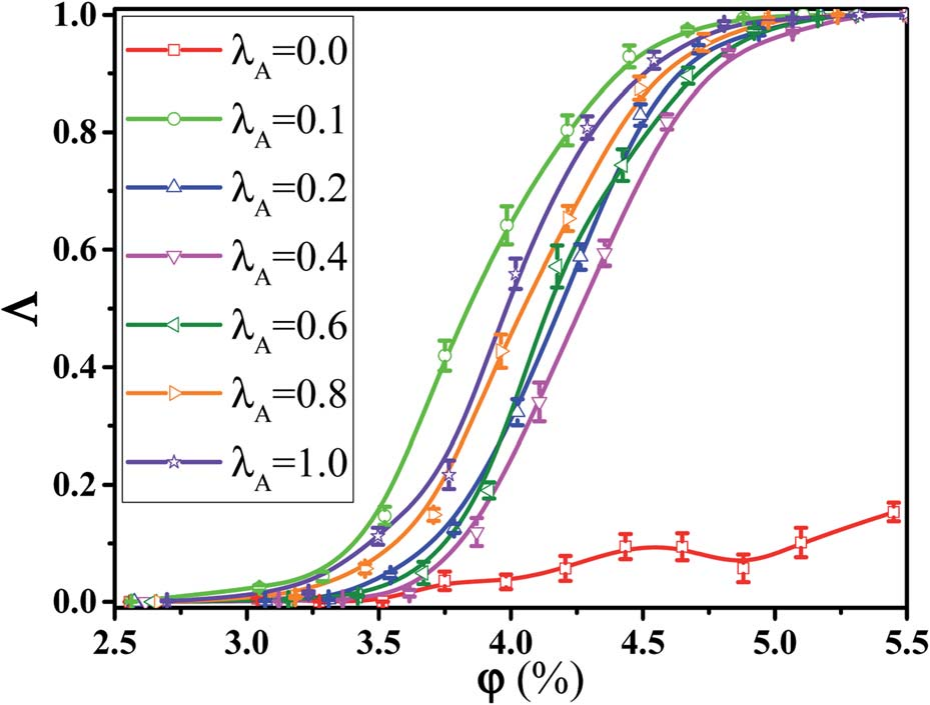
Conductive probability *Λ* as a function of the nanorod (NR) volume fraction *φ* for different NR functionalization degree *λ*_A_. (T* = 1.0, *γ̇* = 0.0).

**Fig. 2:**
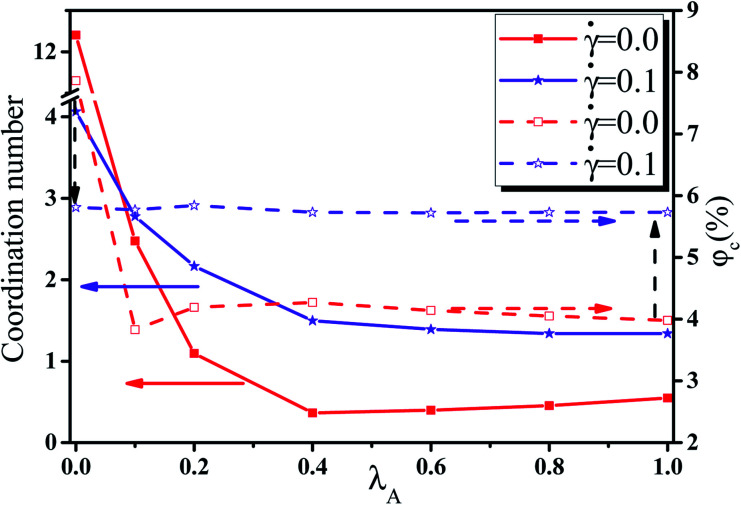
The left axis denotes the coordination number, while the right axis represents the percolation threshold *φ*_c_ with respect to the nanorod functionalization degree *λ*_A_ at shear rate *γ̇* = 0.0 and 0.1. (T* = 1.0).

**Fig. 3:**
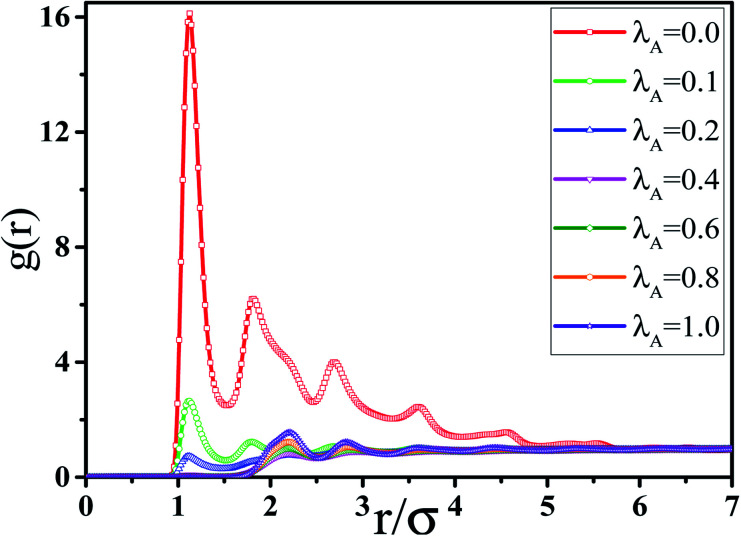
RDF of nanorods (NR) for different NR functionalization degree *λ*_A_. (T* = 1.0, *φ* = 4.0%).

In this section, we intended to investigate the effect of the NR functionalization degree *λ*_A_ on the conductive probability *Λ* under the quiescent state. In these systems, there are three kinds of beads (polymer bead, the functionalized A beads and the unfunctionalized B beads on NR). The number of polymer chains is 1400 for each system. Depending on the NR volume fraction *φ* from 2.55% to 5.45%, the number of NRs varies from 2500 to 5500. Corresponding to it, the box length varies from 37.1*σ* to 37.8*σ*. Fig. 1 presents the change of *Λ* with the *φ* for different *λ*_A_ which exhibits a typical percolation phenomenon. In the percolated region, the conductive network is formed but is not completely developed. Thus, tiny variation of the NR concentration can obviously change the conductive network. Here, the percolation threshold *φ*_c_ is defined as *φ* at *Λ* = 0.5 (ref. 44) which is shown in Fig. 2. It clearly presents that *φ*_c_ first quickly decreases, then increases and last slightly decreases again with increasing *λ*_A_ which exhibits an anti N-type. To explain it, we first characterized the dispersion state of NRs by calculating the inter-NR radial distribution function (RDF) for different *λ*_A_ which is shown in Fig. 3. In general, the peak at *r* = 1*σ* stands for the direct contact aggregation of NRs. The peak at *r* = 2*σ* reflects that NRs aggregates sandwiched by one polymer layer. The depletion effect generating from the polymer chains plays the dominate role at *λ*_A_ = 0.0 which leads to the direct contact aggregation of NRs. This can be proved by the high peak at *r* = 1*σ*. The depletion effect is gradually reduced with the increase of *λ*_A_. Thus, NRs gradually disperse into the matrix which is reflected by the decrease of both peaks at *r* = 1*σ* and *r* = 2*σ*. Especially, the peak at *r* = 1*σ* disappears at *λ*_A_ ≥ 0.4, indicating the absence of the direct contact aggregation of NRs. Meanwhile, the peak at *r* = 2*σ* shows a slight increases which proves that NRs tend to form the aggregation sandwiched *via* one polymer layer. In addition, the coordination number is adopted to denote the dispersion state of NRs which is defined as the average number of NR beads around every NR bead within the distance of *L* = 2.0*σ*.^42^ Small coordination number denotes the uniform dispersion of NR. Fig. S2(a)† shows that the coordination number first gradually decreases and then slightly increases with increasing *λ*_A_, which reflects the most uniform dispersion of NRs at *λ*_A_ = 0.4. It is reported that by employing the integral equation theory, NRs exhibit the contact aggregation, dispersion, bridging, and tele-bridging behavior with tuning the polymer-NR interaction.^45^ In our systems, NRs mainly form the contact aggregation and bridging structures at *λ*_A_ ≤ 0.2, while NRs mainly form the bridging and tele-bridging structures at *λ*_A_ ≥ 0.4. To intuitively observe the NR dispersion state, the snapshots are shown in Fig. S2(b)† for different *λ*_A_. NRs are self-assembled to form the local order structure with NRs aligned side-by-side at *λ*_A_ = 0.0. With the increase of *λ*_A_, the strong interaction between the polymer and NRs induces the dispersion of NRs into the matrix. To better characterize it, the local order of the NR aggregation is calculated as a function of their distance between any two NRs, defined as the second Legendre polynomial 〈*P*_2_(*r*)〉, given by

**Fig. 4:**
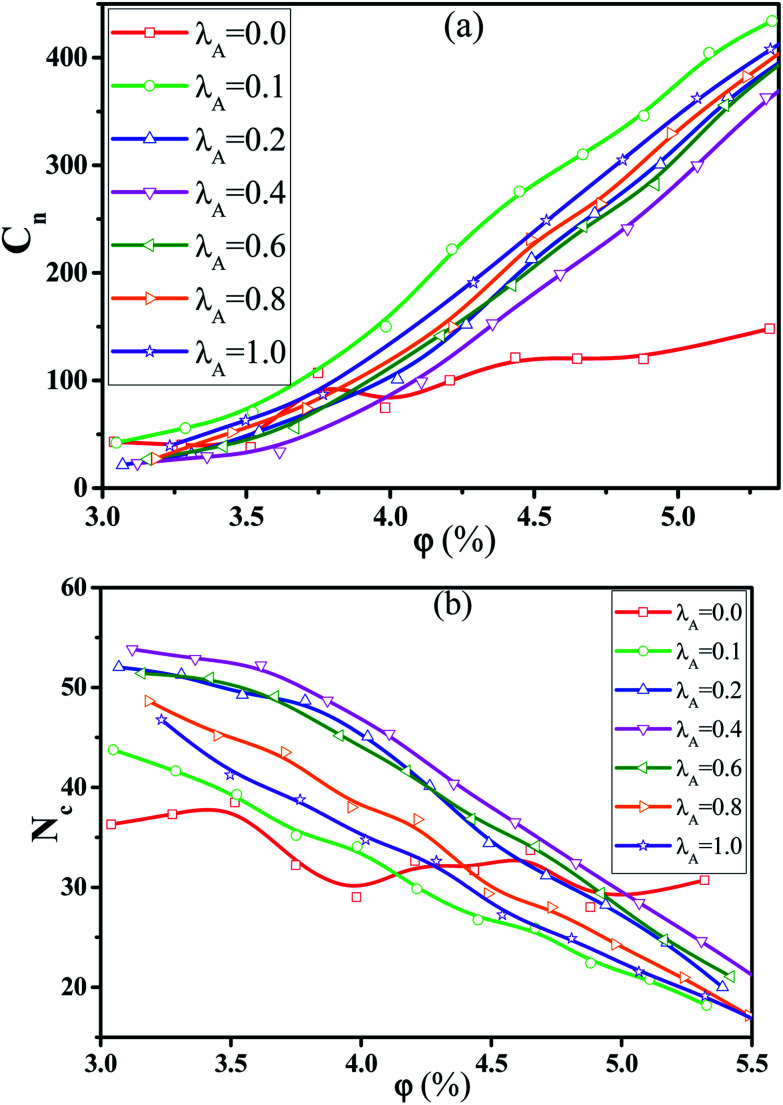
Change of (a) the main cluster size *C*_*n*_ and (b) the total number of clusters *N*_c_ as a function of the nanorod (NR) volume fraction *φ* for different NR functionalization degree *λ*_A_. (T* = 1.0, *γ̇* = 0.0).

**Fig. 5:**
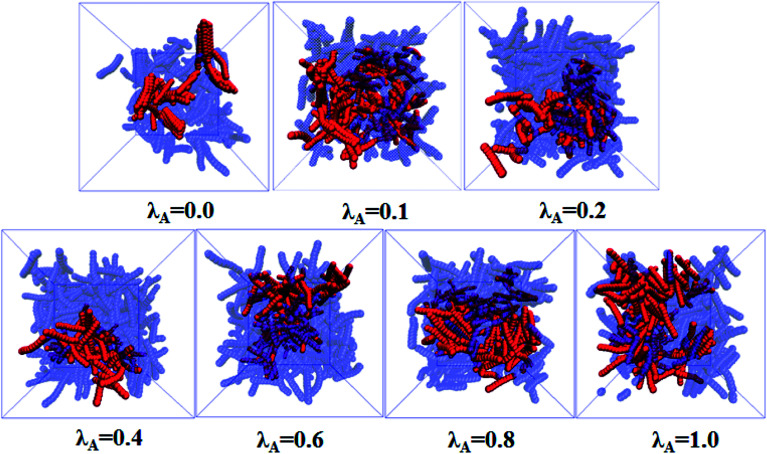
Snapshots of the nanorod (NR) with different NR functionalization degree *λ*_A_ where the polymer chains are neglected for clarity at *φ* = 4.0%. The red spheres denote the NRs within the main cluster. The blue spheres are the other NRs. (T* = 1.0, *γ̇* = 0.0).

4 〈*P*_2_(*r*)〉 = (3〈cos^2^ *θ*〉 − 1)/2

where *θ* denotes the angle between the two end-to-end vectors of a pair of NRs. Averaged over a set of NR pairs, this value is −0.5 for perpendicular alignment, 1.0 for parallel alignment, and 0.0 for random alignment. The local order of the NR aggregation is evaluated in Fig. S3(a).† It reveals very strong orientational correlations for *λ*_A_ = 0.0 which persists over relatively large distances. However, with increasing *λ*_A_, the peaks at *r* < 4*σ* gradually decrease which shows a weak orientational correlations and random distribution of NRs. Then we calculated the number of the nearest neighbor NRs surrounding one NR at a separation closer than 1.5*σ*, denoted by *N*_num_. The probability distribution *P*_*N*_ of the *N*_num_ is obtained for different *λ*_A_ in Fig. S3(b).† There are several peaks (such as *N*_num_ = 3, 4 and 5) of *P*_*N*_ at *λ*_A_ = 0.0. These peaks actually prove the local order of the NR aggregation structure. The peak value of *P*_*N*_ increases with the increase of *λ*_A_. Meanwhile, the *N*_num_ at the maximum *P*_*N*_ gradually shifts from 4 to 0. These results further reflect the breakage of the local order aggregation structure. In summary, NRs are self-assembled to form the direct contact aggregation at *λ*_A_ = 0.0 which is difficult to form the conductive network in the matrix. With the increase of *λ*_A_, the aggregation structure of NRs is broken down which induces their relatively uniform dispersion. This structure transition is favorable to form the conductive network which thus reduces *φ*_c_. Then, the dispersion of NRs is very uniform at *λ*_A_ = 0.4 in Fig. S2(b).† Due to the steric effect of the chains, NRs are considerably far to form conductive network which induces an increase in *φ*_c_. At high *λ*_A_ > 0.4, NRs tend to form a local bridging structure sandwiched *via* one polymer layer, which helps to form a tightly connected conductive network. This induces a decrease in *φ*_c_ again. Furthermore, the main cluster size *C*_*n*_ (the number of NRs within the biggest cluster) and the total number of clusters *N*_c_ are calculated to analyze the conductive network.^46^ In general, the conductive network is easy to be formed in the system with higher *C*_*n*_ and smaller *N*_c_. As shown in Fig. 4(a), within the percolation region, *C*_*n*_ first increases, then decreases and last increases again with increasing *λ*_A_, which is consistent with *Λ*. The system with the maximum *C*_*n*_ shows the minimum *φ*_c_ at *λ*_A_ = 0.1. Compared with *C*_*n*_, *N*_c_ shows an opposite trend in Fig. 4(b). Then, we presented some typical snapshots of the main cluster (red beads) to observe the NR conductive network for different *λ*_A_ at *φ* = 4.0% in Fig. 5. It clearly indicates that the cluster is isolated and *C*_*n*_ is very small at *λ*_A_ = 0.0 which can not form the conductive network. Then *C*_*n*_ increases quickly with the increase of *λ*_A_ = 0.1. Then, *C*_*n*_ gradually decreases with increasing *λ*_A_ to 0.4. Last, *C*_*n*_ shows a slightly increase again with further increasing *λ*_A_. These results are consistent with the conductive probability for different *λ*_A_.

#### 3.1.2 Effect of the interaction *ε*_pA_ between polymer and A beads

**Fig. 6:**
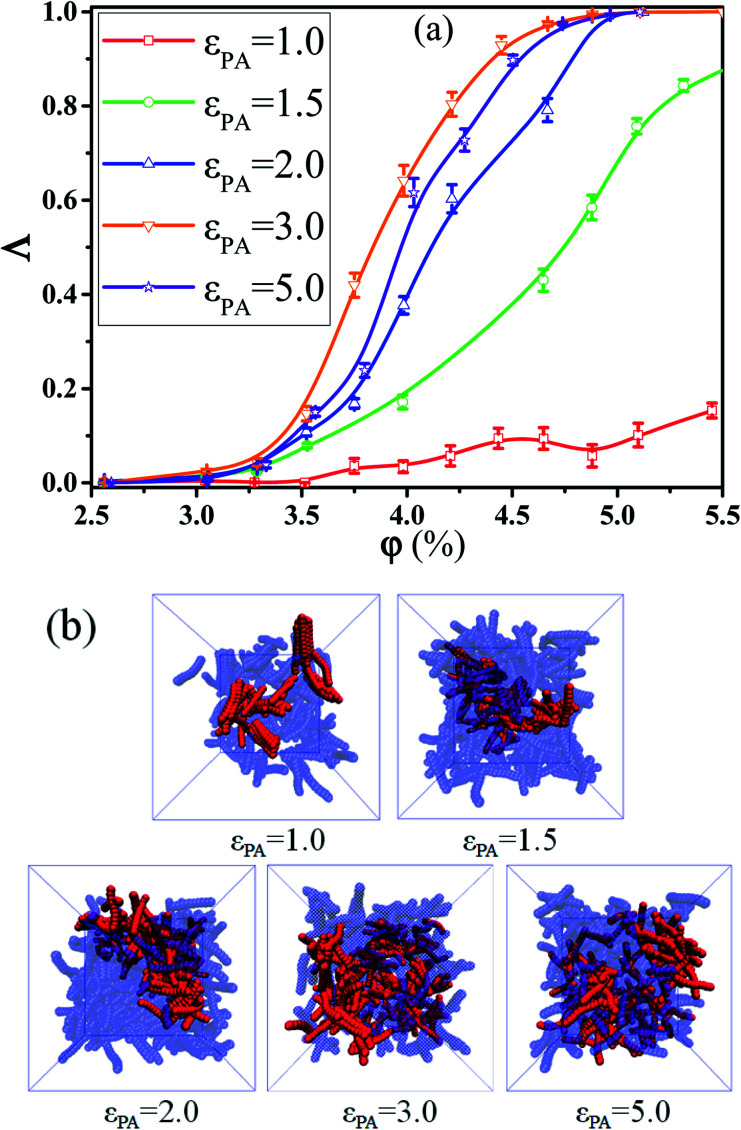
(a) Conductive probability *Λ* as a function of the nanorod (NR) volume fraction *φ* and (b) snapshots of NRs with different interaction *ε*_pA_ between polymer and A beads where the polymer chains are neglected for clarity at *φ* = 4.0%. The red spheres denote the NRs within the main cluster. The blue spheres are the other NRs. (T* = 1.0, *γ̇* = 0.0).

Next, we intended to investigate the effect of the interaction *ε*_pA_ between polymer and A beads on the conductive probability *Λ* by fixing the *λ*_A_ = 0.1 where the system exhibits the maximum conductive probabilities. The dependence of the *Λ* on the *φ* is shown in Fig. 6(a) for different *ε*_pA_. It is found that *Λ* first increases and then decreases with the increase of the *ε*_pA_ which reaches the maximum value at mediate *ε*_pA_ = 3.0. Corresponding to *Λ*, the percolation threshold *φ*_c_ shows a contrary trend with the *ε*_pA_. To understand it, the inter-NR RDF is characterized for different *ε*_pA_ in Fig. S4† which reflects the gradual dispersion of NRs into the matrix. This can be proved by the decrease of the peak at *r* = 1*σ*. Then, the local order 〈*P*_2_(*r*)〉 of the NR aggregation is calculated as a function of their distance between any two NRs in Fig. S5(a).† The results present that the local order of the NR aggregation is gradually broken down with the increase of *ε*_pA_ which is indicated by the decrease of peaks at *r* < 4*σ*. Similarly, we characterized the probability distribution *P*_*N*_ of the *N*_num_ for different *ε*_pA_ in Fig. S5(b).† The peak value of *P*_*N*_ increases with the *ε*_pA_. Meanwhile, the *N*_num_ at the maximum *P*_*N*_ gradually shifts from 4 to 1. These results further prove the observed results above. In summary, NRs form the direct contact aggregation at *ε*_pA_ = 1.0 which leads to high *φ*_c_. With the increase of *ε*_pA_ (1.0 < *ε*_pA_ ≤ 3.0) the dispersion state of NRs changes from direct aggregation to a relative dispersion. This can benefit the formation of the conductive network which reduces *φ*_c_. With further increasing *ε*_pA_ ≥ 5.0, single NRs (high *P*_*N*_ at *N*_num_ = 0.0) cannot effectively form a conductive network because of the large distances between them. This induces an increase in *φ*_c_. Furthermore, the main cluster size *C*_*n*_ is calculated for different *ε*_pA_ in Fig. S6.† It is found that *C*_*n*_ first increases and then decrease with the increase of *ε*_pA_ which is consistent with *Λ*. Last, the snapshots of the main cluster (red beads) are presented for different *ε*_pA_ at *φ* = 4.0% in Fig. 6(b) which further rationalizes the observed results. In total, the PNCs exhibit the maximum conductive probability at the mediate *ε*_pA_ = 3.0.

#### 3.1.3 Effect of the external shear field

The external shear field is a very important method for manufacturing PNCs which will affect the electrical conductivity.^5^ The original conductive network will be broken down under the shear field. Then a new conductive network will be formed. It is very necessary to understand how the shear field affects the conductive network for different *λ*_A_.^9,47^ Here, three kinds of conductive probabilities, namely *Λ*, *Λ*_‖_ and *Λ*_⊥_ are considered which are explained in Section 2. Here, the SLLOD equations are adopted to realize the shear simulation, which is one of the most widely used methods for studying the shearing systems.^48^ The special Lees–Edwards “sliding brick” boundary conditions is used for the SLLOD method which is very effective for the shear simulation.^49^ The shear field is exerted on the simulation box by moving top *xy* plane of the simulation box along the *x* direction.

**Fig. 7:**
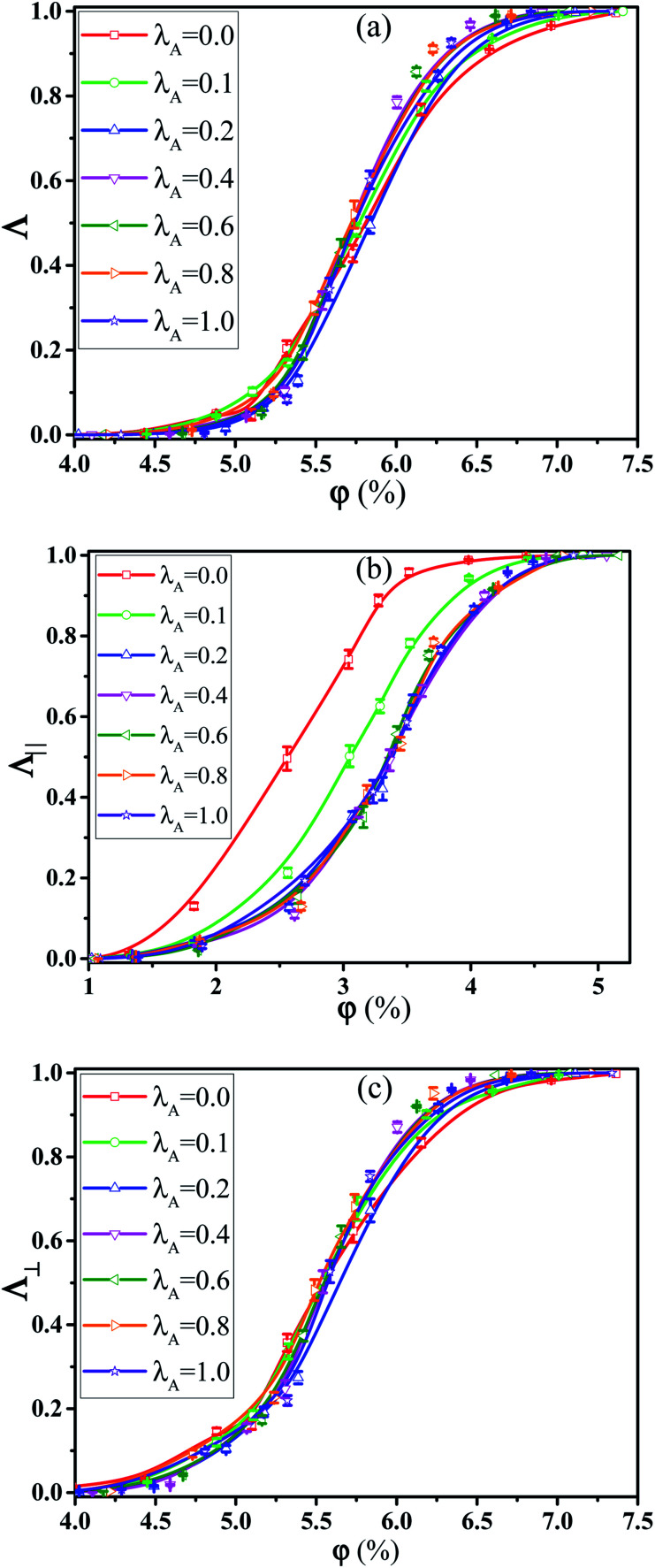
(a) Homogeneous conductive probability *Λ*, (b) directional conductive probability *Λ*_‖_ parallel to the shear direction, and (c) directional conductive probability *Λ*_⊥_ perpendicular to the shear direction as a function of the nanorod (NR) volume fraction *φ* for different NR functionalization degree *λ*_A_. (T* = 1.0, *γ̇* = 0.1).

**Fig. 8:**
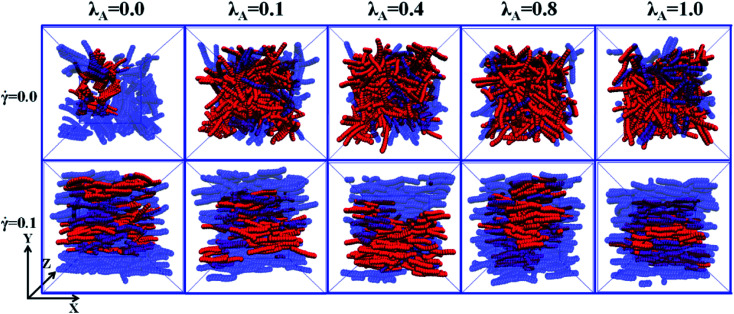
Some typical snapshots of the nanorods (NR) with different NR functionalization degree *λ*_A_ at *γ̇* = 0.0 and 0.1. The red spheres denote the NRs within the main cluster. The blue spheres are the other NRs. *X* direction is the shear direction. (T* = 1.0, *φ* = 5.5%).

First, we investigated the effect of the NR functionalization degree *λ*_A_ on the conductive probability of PNCs by fixing the shear rate = 0.1. Fig. 7 presented the dependence of *Λ*, *Λ*_‖_, and *Λ*_⊥_ on *λ*_A_for different *φ* in a steady shear flow. To clearly present it, the percolation threshold *φ*_c_ of PNCs at *γ̇* = 0.1 is obtained in Fig. 7(a) and shown in Fig. 2 for different *λ*_A_. It is interesting to find that the *φ*_c_ is nearly independent of the *λ*_A_ at *γ̇* = 0.1 (namely the similar *Λ*), which is different with *φ*_c_ at *γ̇* = 0.0 in Fig. 2. This is actually consistent with the experimental result.^50^ To better understand it, the *φ*_c_ at *γ̇* = 0.0 and 0.1 are presented together in Fig. 2. Then, we characterized how the shear field changes the conductive network. First, the inter-NR RDF under the shear field is calculated for different *λ*_A_ in Fig. S7.† Compared with those at *γ̇* = 0.0 in Fig. 3, the peaks at *r* = 1*σ* and 2*σ* are found to gradually decrease for *λ*_A_ = 0.0 at *γ̇* = 0.1, which indicates the breakage of the direct contact aggregation of NRs. Thus, the shear field induces the decrease in *φ*_c_ for *λ*_A_ = 0.0. However, they show an increase for *λ*_A_ ≥ 0.1 which reflects the breakage of the original conductive network. As a result, this leads to the increase in *φ*_c_ for *λ*_A_ ≥ 0.1 under the shear field. Next, the local order of the NR aggregation 〈*P*_2_(*r*)〉is presented in Fig. S8(a).† Similarly, compared with those at *γ̇* = 0.0 in Fig. S3(a),† the fluctuation of 〈*P*_2_(*r*)〉 is very small at *r* < 3*σ* at *γ̇* = 0.1, especially for *λ*_A_ = 0.0. This also indicates that the shear field induces the breakage of the direct contact aggregation of NRs. Meanwhile, the orientation of NRs along the shear direction is responsible for the non-zero value of 〈*P*_2_(*r*)〉 at long distance *r*. In addition, the number of the nearest neighbor NRs surrounding one NR (*N*_num_) is calculated. From Fig. S8(b),† compared with that at *γ̇* = 0.0 in Fig. S3(b),† the *N*_num_ at the maximum probability distribution *P*_*N*_ decreases for *λ*_A_ = 0.0 at *γ̇* = 0.1. However, the *P*_*N*_ decreases at *N*_num_ = 0 while it increases at *N*_num_ = 1. These results indicate that the shear field exerts a significant effect on the conductive network. To better explain it, we turned to *Λ*_‖_ and *Λ*_⊥_, which determine the *Λ*. It is noted that two directional conductive probabilities along the gradient direction and the vorticity direction are averaged as the directional conductive probability perpendicular to the shear direction *Λ*_⊥_ because they are very similar. As shown in Fig. 7(b), *Λ*_‖_ first shows a decrease with *λ*_A_ from 0.0 to 0.1 and then keeps unchanged with *λ*_A_, which is generally related to both the dispersion state and the orientation of NRs. Thus, we characterized the orientation degree of NRs for different *λ*_A_, which is described by the second-order Legendre polynomials 〈*P*_2_〉. It is given by 〈*P*_2_〉 = (3 〈cos^2^ *θ*〉 − 1)/2, where *θ* denotes the angle between the end-to-end vector of NRs and the shear direction. As shown in Fig. S9,† it is found that the 〈*P*_2_〉 first increases and then keeps nearly unchanged with the increase of *λ*_A_, which is consistent with *Λ*_‖_. In addition, it is noted that the *P*_N_ at *N*_num_ = 3 or 4 for *λ*_A_ = 0.0 and 0.1 is larger than that for *λ*_A_ ≥ 0.2 in Fig. S8(b).† This indicates that more NRs connect 3 to 4 other NRs for *λ*_A_ = 0.0 and 0.1, which can help to form the conductive network along the shear direction. It is noted than *Λ*_‖_ is larger than *Λ*_⊥_. Thus, *Λ* strongly depends on *Λ*_⊥_, rather than *Λ*_‖_. From Fig. 7(c), *Λ*_⊥_ exhibits a similar trend with *Λ*. Similarly, we calculated the main cluster size *C*_*n*_ to analyze the conductive network in Fig. S10.† The results indicate that *C*_*n*_ is nearly similar for different *λ*_A_ which is consistent with *Λ*. To intuitively observe the NR conductive network, Fig. 8 presents the snapshots of some typical systems (*φ* = 5.75%) with different *λ*_A_. It clearly shows that NRs gradually align along the shear direction. Meanwhile, *C*_*n*_ (the number of red beads) is similar for different *λ*_A_. In summary, the original conductive network is broken down and a new one forms under the shear field. Compared with under the quiescent state, the breakage of the direct contact aggregation structure of NRs enhances *Λ* and reduces *φ*_c_ for *λ*_A_ = 0.0 under the shear field; However, *φ*_c_ increases for *λ*_A_ ≥ 0.1. Thus, compared with under the quiescent state, the decrease or the increase of the conductive probability depends on the initial dispersion state of NRs under the shear field, which can be tuned by *λ*_A_.

**Fig. 9:**
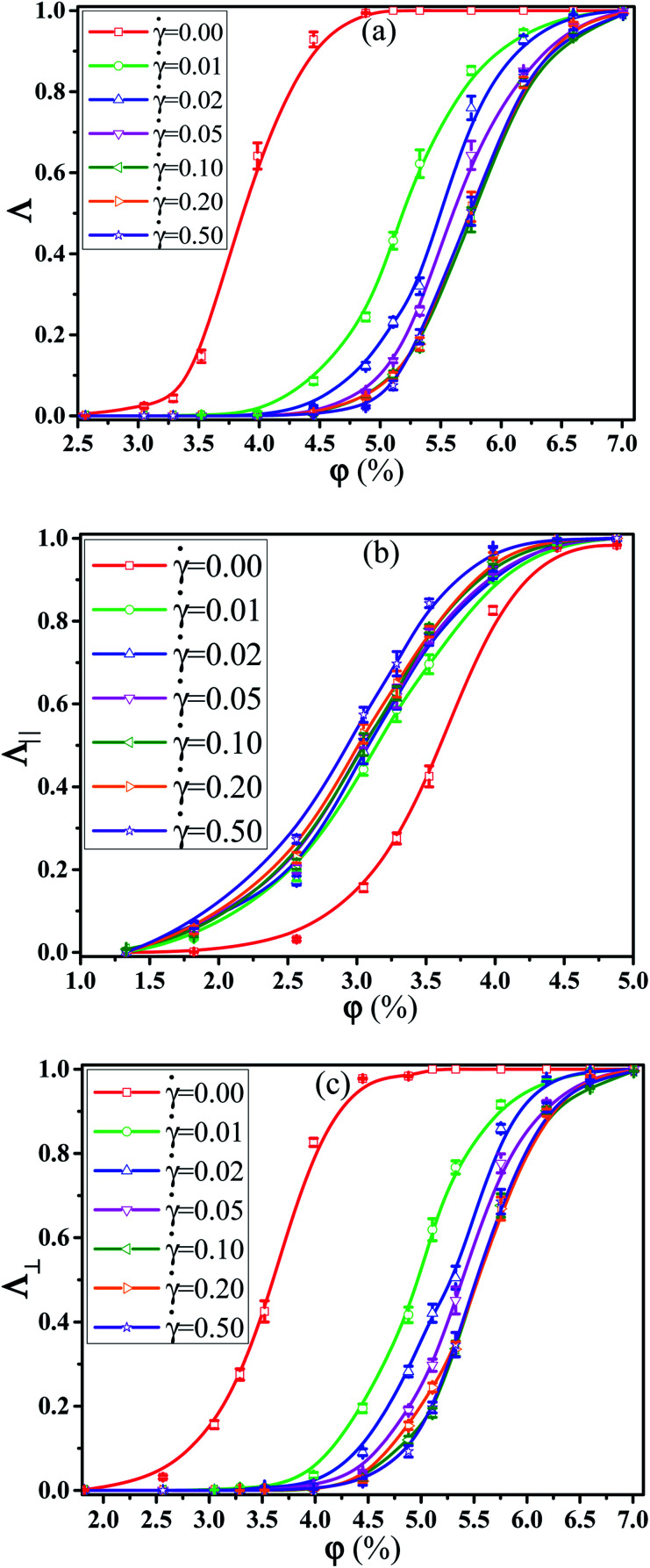
Homogeneous conductive probability *Λ*, (b) directional conductive probability *Λ*_‖_ parallel to the shear direction, and (c) directional conductive probability *Λ*_⊥_ perpendicular to the shear direction as a function of the nanorod volume fraction *φ* for different shear rate *γ̇*. (T* = 1.0, *λ*_A_ = 0.1).

**Fig. 10:**
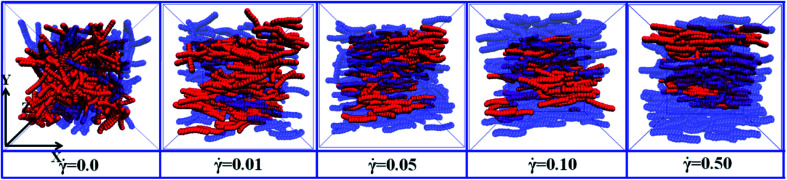
Some typical snapshots of the nanorods (NR) with different shear rate *γ̇*. The red spheres denote the NRs within the main cluster. The blue spheres are the other NRs. *X* direction is the shear direction. (T* = 1.0, *φ* = 5.5%, *λ*_A_ = 0.1).

At last, the effect of the shear rate *γ̇* on the conductive probability of PNCs is investigated at *λ*_A_ = 0.1 where the system shows the highest conductive probability under the quiescent state. Fig. 9 presents the change of *Λ*, *Λ*_‖_, and *Λ*_⊥_ with *γ̇*. From Fig. 9(a), it is found that the *Λ* first decreases and then keeps unchanged with the increase of *γ̇*, which indicates the breakage of the conductive network under the shear field. As shown in Fig. S11,†*φ*_c_ increases slowly from 3.85% to 5.75% when *γ̇* varies from 0.0 to 0.1. Then, it keeps nearly unchanged with further increasing *γ̇* to 0.5. This is because the shear field destroys the conductive network which is reflected by the decrease of *C*_*n*_ in Fig S12.† From Fig. 9(b), *Λ*_‖_ exhibits a continuous increase with *γ̇* from 0.0 to 0.5, which is attributed to the high orientation degree of NRs in Fig. S13† and the relatively dispersion structure of NRs in Fig. S14.†*Λ*_⊥_ shows a similar trend with *Λ* as the increase of *γ̇*. The significant decrease of the *λ*_⊥_ is attributed to the breakage of the inter-NR connection perpendicular to the shear direction. At last, we presented the snapshots of the conductive network for different *γ̇* (*φ* = 5.50%) in Fig. 10. It is found that the *C*_*n*_ gradually decreases when *γ̇* increases from 0.0 to 0.1. Then, it nearly keeps unchanged with further increasing *γ̇* which is consistent with the *Λ*. In total, the orientation and the dispersion state of NRs along the shear direction changes with *γ̇* which synergistically affects the conductive network and conductive probability.

## 4. Discussion

When mapping the coarse-grained model to real polymers, the interaction parameter *ε* is about 2.5–4.0 kJ mol^−1^ for the common polymers,^36^ indicating that *ε*_pA_ = 5.0 (in units of *ε*) is about 12.5–20.0 kJ mol^−1^. It has been reported that the interaction strengths of the natural rubber with adsorption sites on carbon black vary from 13 to 35 kJ mol^−1^,^51^ and they are mainly attributed to van der Waals forces. In addition, for the adopted coarse-grained model, the persistence length is about 0.676*σ*. For real polymers, the persistence length varies from 0.35 nm to 0.76 nm,^52^ which indicates that *σ* is roughly about 1 nm. Meanwhile, we have calculated the rotational diffusion coefficient (*D*_r_) of nanorod, which is about 1.42 × 10^−4^*τ*^−1^.^53^ The shear rate *γ̇* is between 0.01*τ*^−1^ and 0.5*τ*^−1^ in our simulation, which is higher than that in the real physical experiments. As a result, the Peclet number 
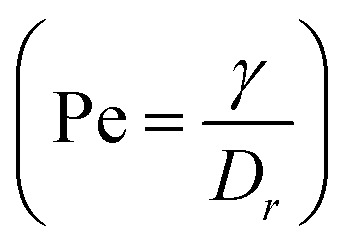
 roughly varies from 70 to 3500, which is comparable with the experimental Peclet numbers.^54^ Thus, our simulation results can reflect the real experiments.

According to the previous works, the electrical conductivity is strongly dependent on the conductive network, which is affected by the polymer–filler interactions,^12^ the aspect ratio,^55^ shape,^56^ flexibility,^23^ and alignment^24^ of filler, *etc.* In this work, we mainly focused on the effect of the NR functionalization on the conductive behavior of PNCs under the quiescent state and under the shear field. In experiments,^57^ the percolation threshold varies from 0.01 vol% to 4.75 vol% in different CNT filled PNCs, which is smaller than our results (from 3.83% to 7.86%). To explain this difference, firstly both the aspect ratio of CNTs and the chain length in experiments are much greater than those in this work because of the limitation of computational power. Secondly, in the simulation, the conductive probability is adopted to qualitatively reflect the electrical conductivity, but not quantitatively. Meanwhile, the conductive probability just changes from 0.0 to 1.0 (one order of magnitude) in the simulation which also depends on the tunneling distance. However, the electrical conductivity can change over more than 10 orders of magnitude near the percolation threshold in experiments.^57^ Therefore, quantitative comparison between the experiments and our simulation is difficult. In addition, some experimental works^58,59^ reported that the chemical functionalization of CNTs increases the compatibility between polymer matrix and CNTs, which, in turn, causes a significant increase in the electrical conductivity. However, it is difficult to decide the optimal functionalization degree of CNT where the PNCs exhibits the maximum electrical conductivity. Meanwhile, the other work^60^ indicated the decrease of the electrical conductivity. In general, the functionalization of CNT breaks the conjugate planar structure which reduces the inherent conductivity of the CNT. However, it is not considered in the current model. Furthermore, the electrical conductivity of PNCs can be obtained by the electrical network model *via* the filler network^24,61,62^ which can be used to further optimize our model in the further. In total, our simulation can roughly reflect the above experimental systems. The main new phenomena revealed here are summarized below: (1) the percolation threshold of PNCs first decreases, then increases and last slightly decreases again with the increase of *λ*_A_, which exhibits an anti N-type under the quiescent state. However, the percolation threshold is nearly independent of the *λ*_A_ under the shear field. (2) Compared with in the quiescent state, the decrease or the increase of the conductive probability under the shear field depends on the *λ*_A_. (3) Even though the dispersion state of NRs is different, the percolation threshold is similar for different *λ*_A_ under the shear field, which indicates that the dispersion state of NRs is not completely correlated to the conductive network.

## 5. Conclusions

In this work, by adopting a coarse-grained molecular dynamics simulation, we investigated the effect of nanorod (NR) functionalization on the conductive probability of PNCs under the quiescent state or under the shear field. The self-attractive NRs form a direct contact aggregation structure with the local order in the matrix. Then, the NR functionalization induces the breakage of the aggregation structure of NRs, which improves their dispersion state. Last a local bridging structure of NRs sandwiched *via* one polymer layer is formed at high functionalization degree *λ*_A_. As a result, the percolation threshold of PNCs first quickly decreases, then increases and last slightly decreases again with increasing *λ*_A_, which exhibits an anti N-type under the quiescent state. Meanwhile, the percolation threshold first decreases and the increase with the increase of the interaction between polymer and the functionalized beads which reaches the lowest value at the moderate interaction. However, it is nearly independent of the *λ*_A_ under the shear field. Compared with in the quiescent state, the shear induces the breakage of the NR aggregation for *λ*_A_ = 0.0 which enhances the conductive probability. However, the conductive network is broken down under the shear field for *λ*_A_ > 0.0 which reduces the conductive probability. Thus, compared with in the quiescent state, the decrease or the increase of the conductive probability under the shear field depends on the *λ*_A_. Interestingly, the dispersion state of NRs is different for different *λ*_A_ under the shear field. However, the conductive probability is similar. This reflects that the dispersion state of NRs is not completely correlated to the conductive network. In summary, this work presents that the NR functionalization is an effective method to tune and control the conductive network, which can help fabricate PNCs with high electrical conductivity.

## Conflicts of interest

There are no conflicts to declare.

## Supplementary Material

RA-009-C9RA04680A-s001
