# Supplementary material for: Percolation analysis of the electrical conductive network in a polymer nanocomposite by nanorod functionalization
Source: RSC Adv. 2019 Nov 7;9(62):36324–33. doi: 10.1039/c9ra04680a (PMC9074929; doi:10.1039/c9ra04680a)
Supplement: RA-009-C9RA04680A-s001 [file RA-009-C9RA04680A-s001.pdf]

Electronic Supplementary Material (ESI) for RSC Advances.
This journal is © The Royal Society of Chemistry 2019

Supporting Information

Percolation analysis of the electrical conductive network in a polymer nanocomposites by nanorod functionalization

Ruibin Ma[1,2], Guangyao Mu[1,2], Huan Zhang,[3] Jun Liu, [1,2] Yangyang Gao[1,2*], Xiuying Zhao[1,2*], Liqun Zhang[1, 2 *]

[1]Key Laboratory of Beijing City on Preparation and Processing of Novel Polymer Materials, Beijing University of Chemical Technology, 10029, China

[2] State Key Laboratory of Organic-Inorganic Composites, Beijing University of Chemical Technology, 100029, China

[3]Aerospace Research Institute of Materials and Processing Technology, Beijing, 100076, China

* Corresponding author: gaoyy@mail.buct.edu.cn or zhaoxy@mail.buct.edu.cn or zhanglq@mail.buct.edu.cn

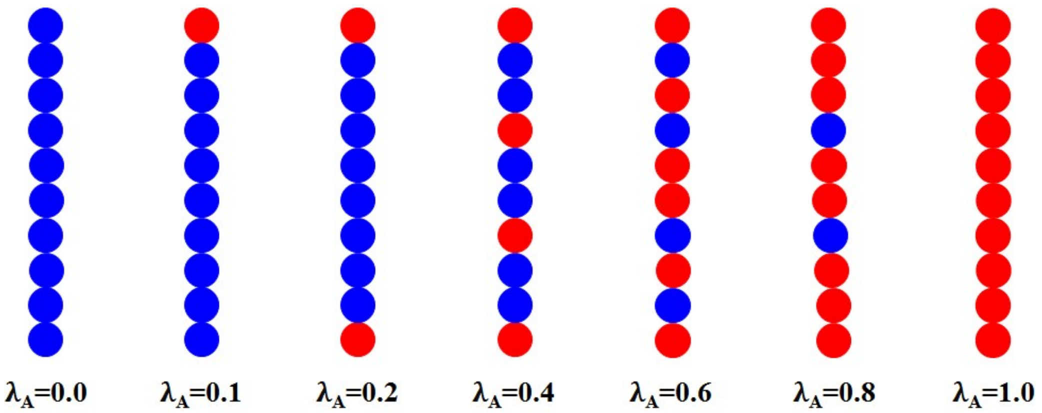

Fig. S1 The distribution of A and B beads in each nanorod (NR) at different NR functionalization degree λ_A. The red spheres denote the A beads and the blue spheres denote the B beads.

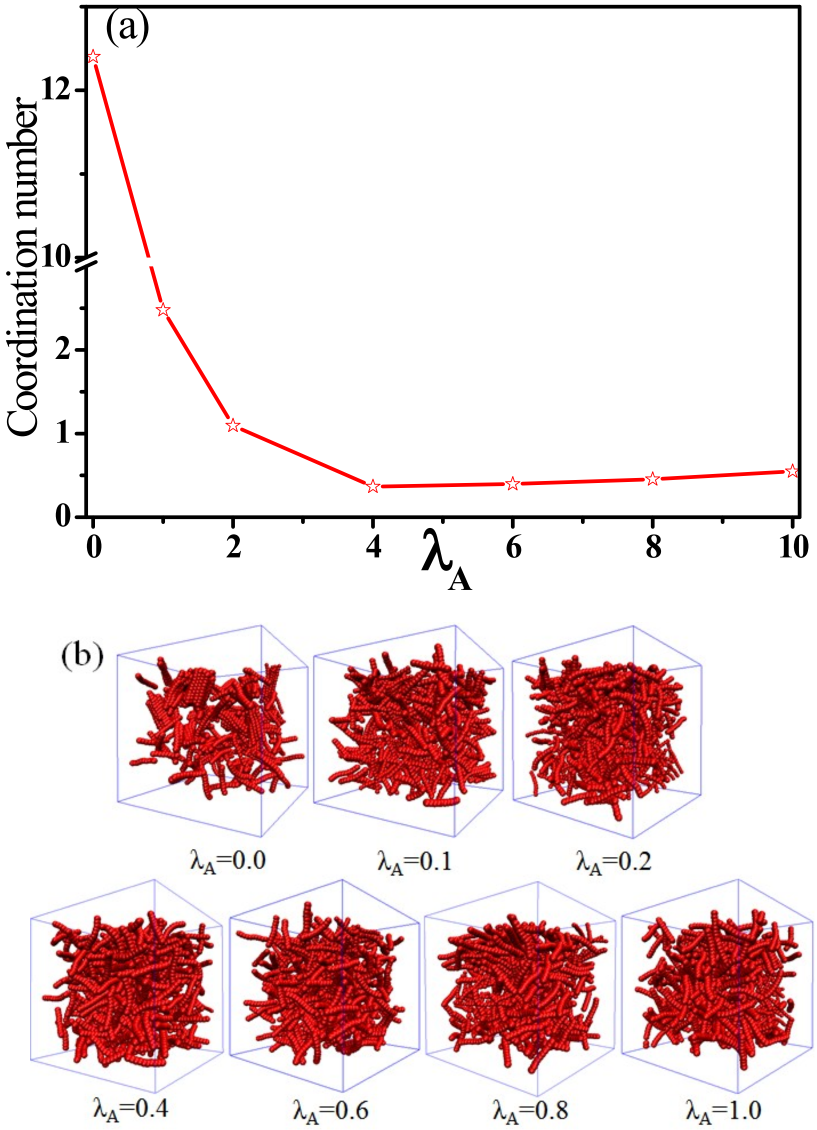

Fig. S2(a) The coordination number and (b) snapshots for different nanorods (NR) functionalization degree λ_A where the polymer chains are neglected for clarity and the red spheres denote the NRs. (T^*=1.0, φ=4.0%)

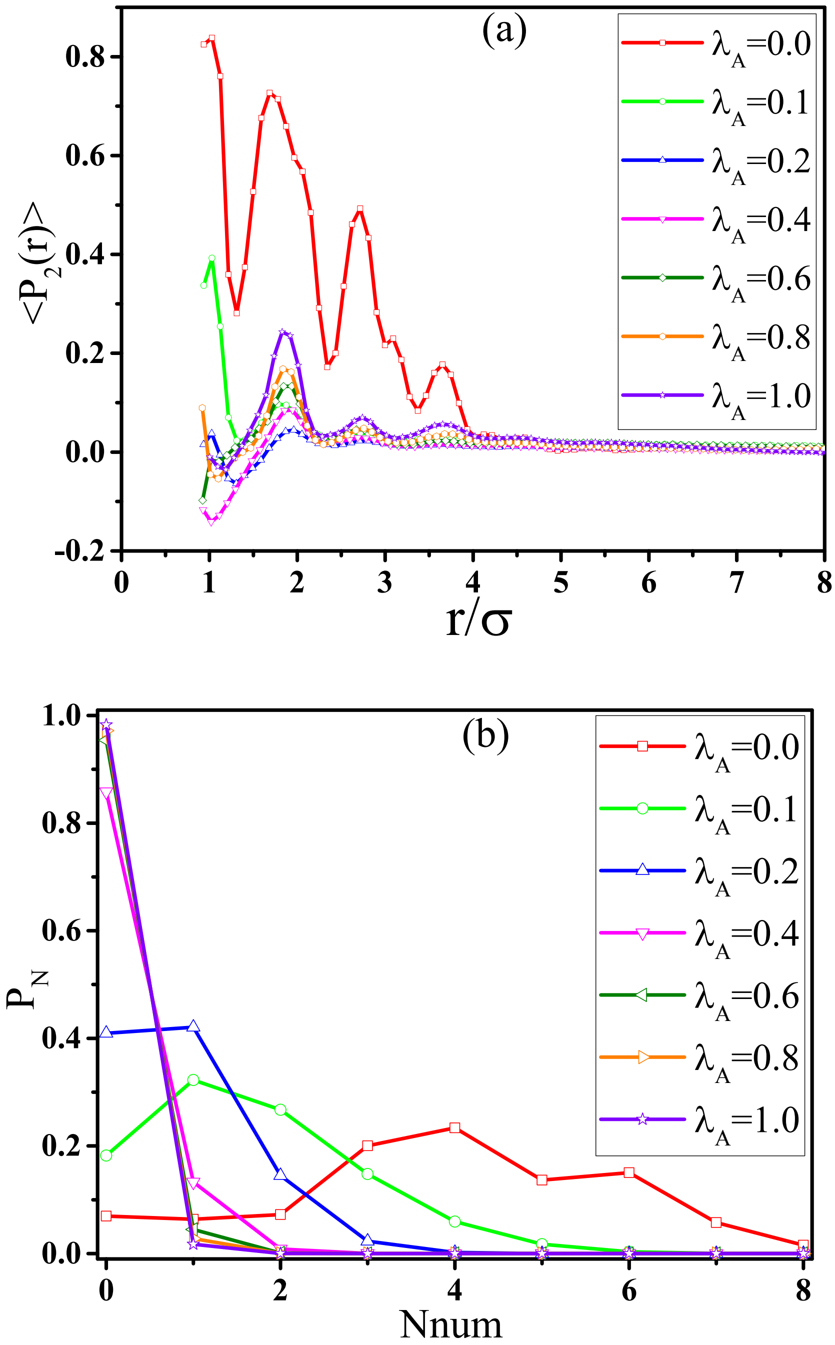

Fig. S3(a) The local order structure $<P_2(\mathrm{r})>$ of the nanorod (NR) aggregation and (b) the probability distribution (P_N) of the nearest neighbor NRs surrounding one NR at a separation closer than 1.5σ (Nnum) for different NR functionalization degree λ_A. (T^*=1.0, φ=4.0%, $\dot{\gamma}$=0.0)

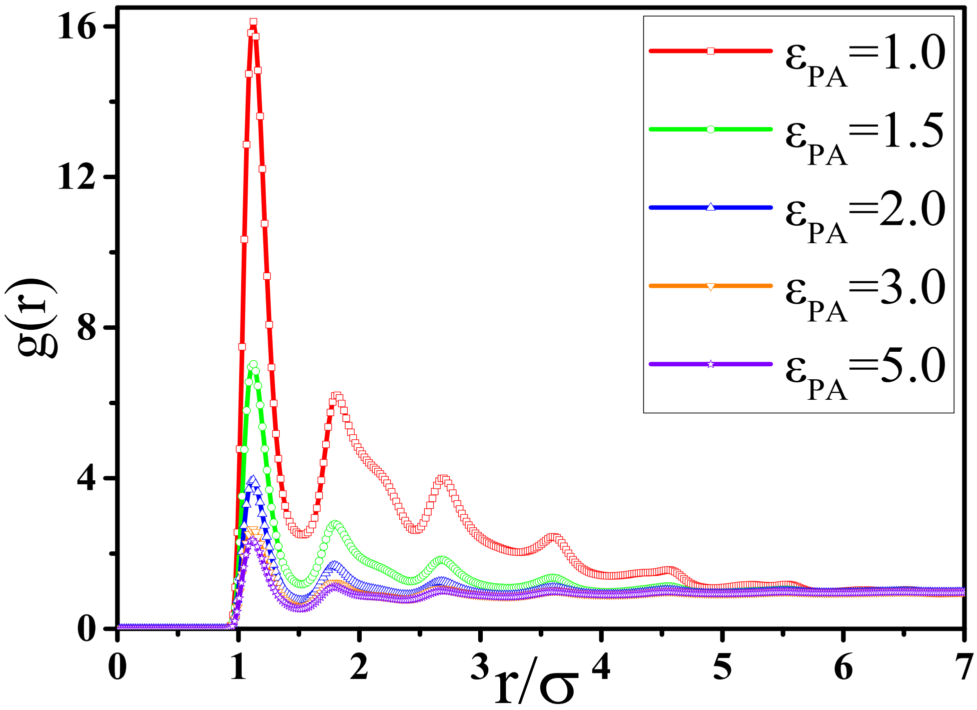

Fig. S4 RDF of nanorods for different interaction ε_{pA} between polymer and A beads. (T^*=1.0, φ=4.0%)

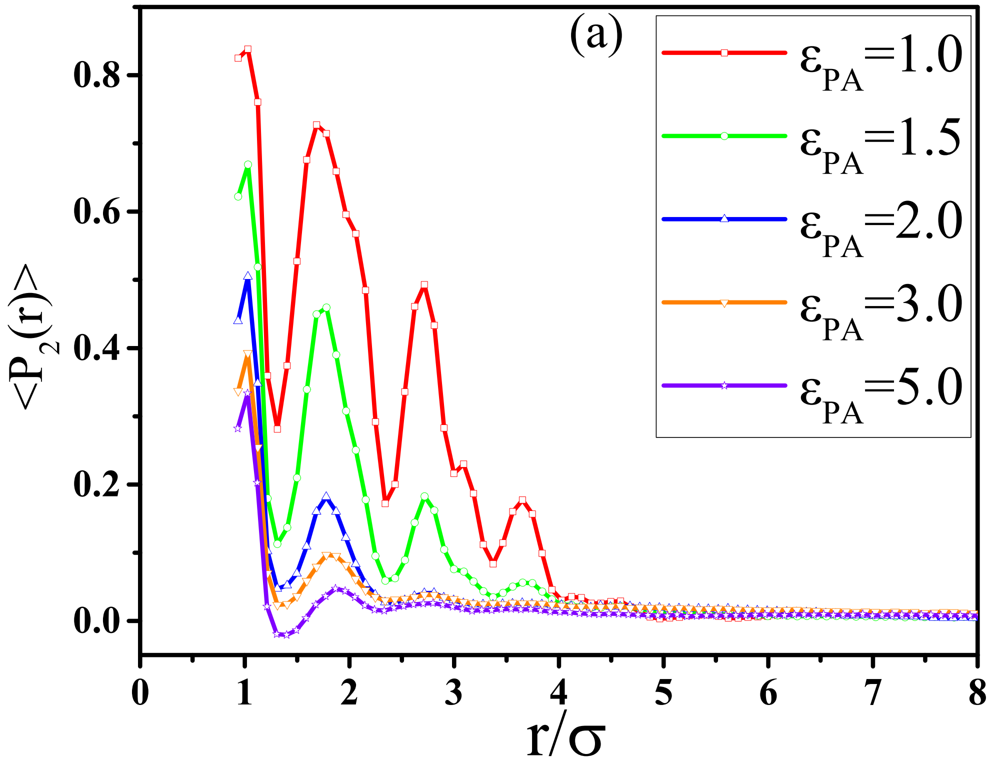

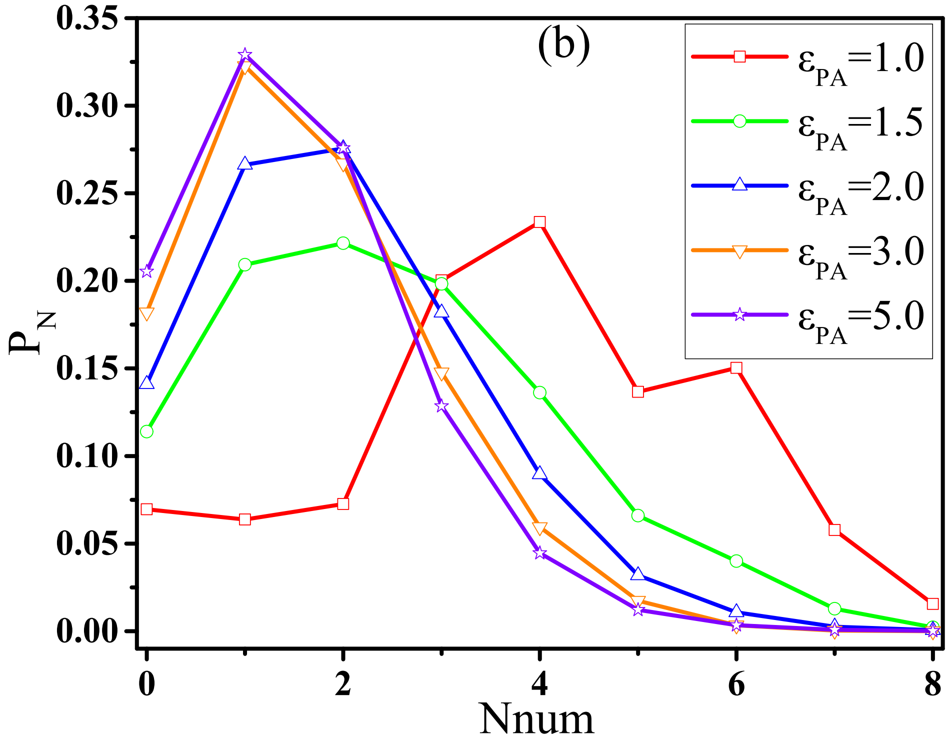

Fig. S5(a) The local order structure $< P_2(\mathrm{r}) >$ of the nanorod (NR) aggregation and (b) the probability distribution (P_N) of the nearest neighbor NRs surrounding one NR at a separation closer than 1.5σ (Nnum) for different interaction ε_{pA} between polymer and A beads. (T^* =1.0, φ =4.0%, $\dot{\gamma}$=0.0)

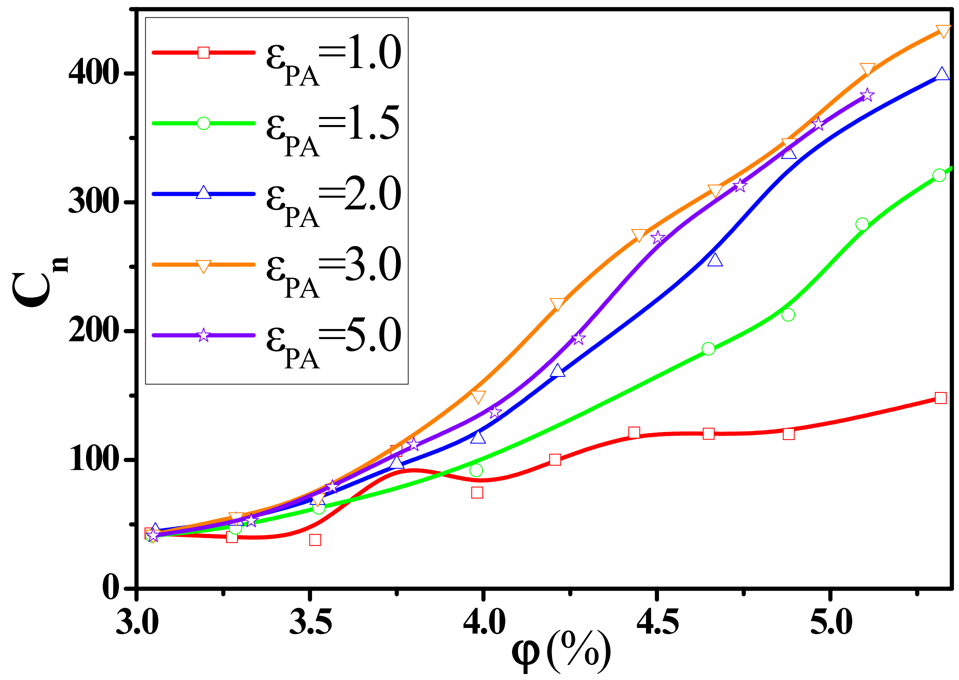

Fig. S6 Change of the main cluster size C_n as a function of the nanorod (NR) volume fraction φ for different interaction ε_{pA} between polymer and A beads. (T^* =1.0, $\dot{\gamma}$=0.0)

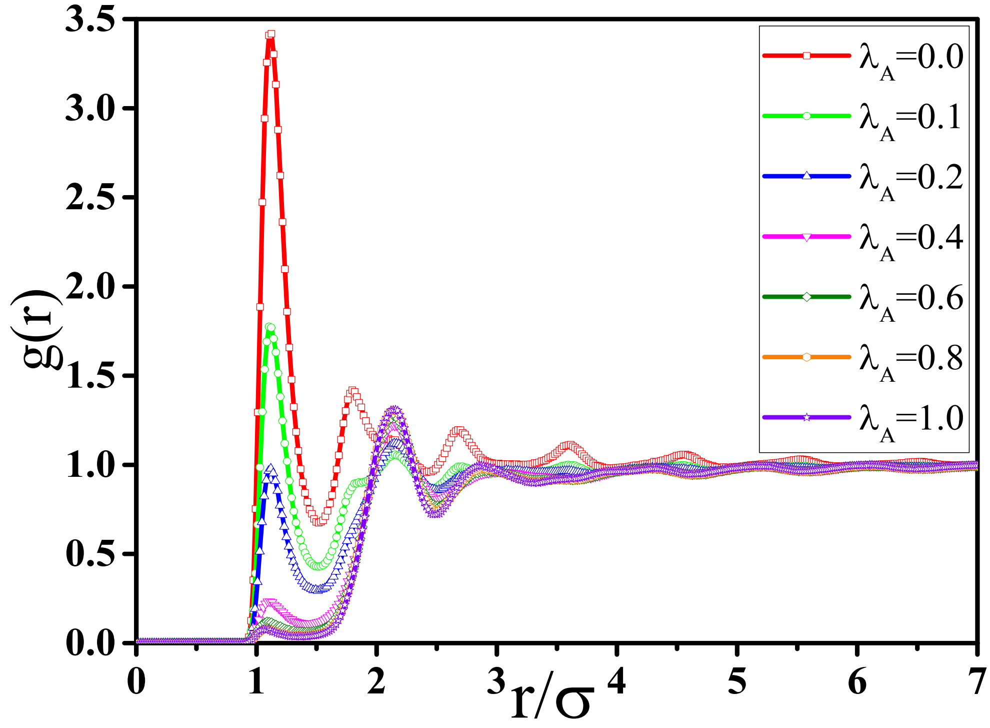

Fig. S7 RDF of nanorods (NR) for different NR functionalization degree λ_A. (T^*=1.0, φ=4.0%, $\dot{\gamma}$=0.1)

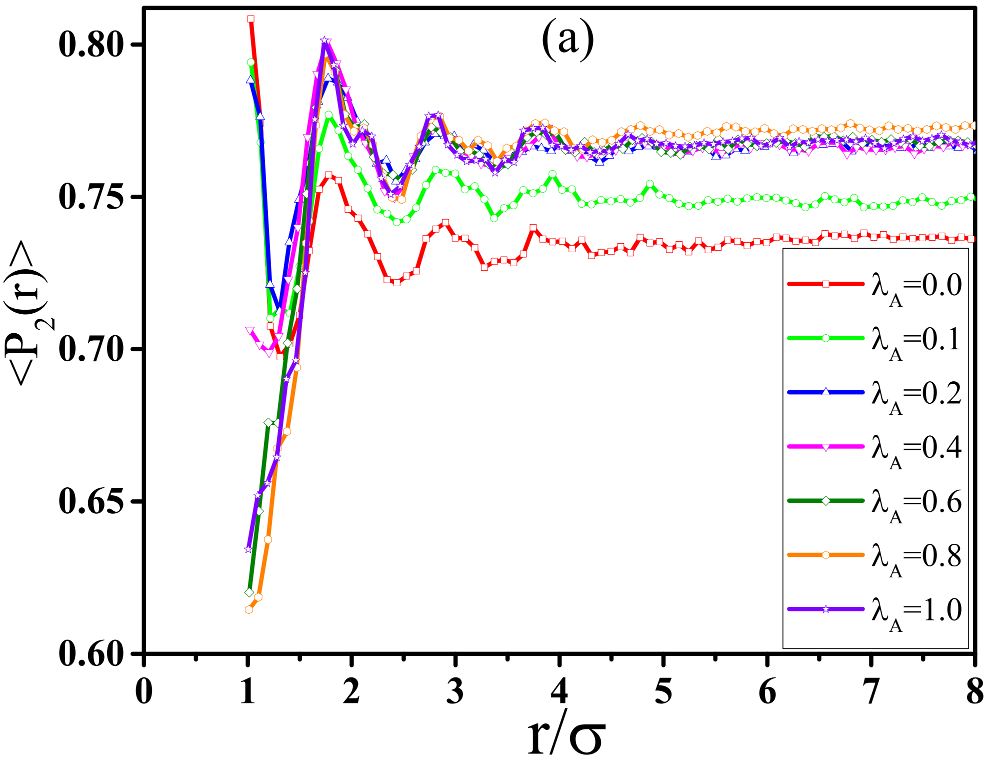

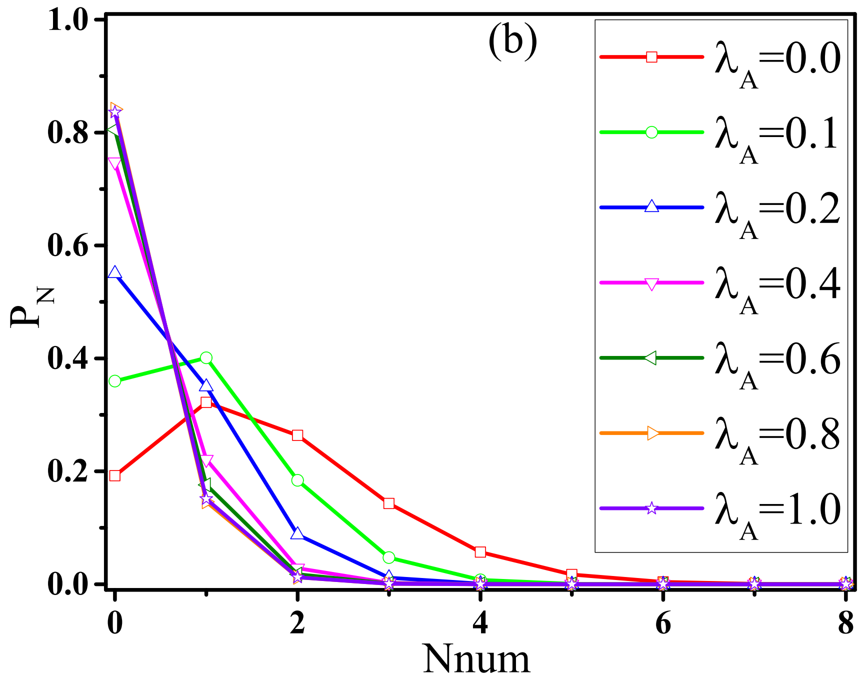

Fig. S8(a) The local order structure $< P_2(\mathrm{r}) >$ of the nanorod (NR) aggregation and (b) the probability distribution (P_N) of the nearest neighbor NRs surrounding one NR at a separation closer than 1.5σ (Nnum) for different NR functionalization degree (λ_A).(T^*=1.0, φ=4.0%, $\dot{\gamma}$=0.1)

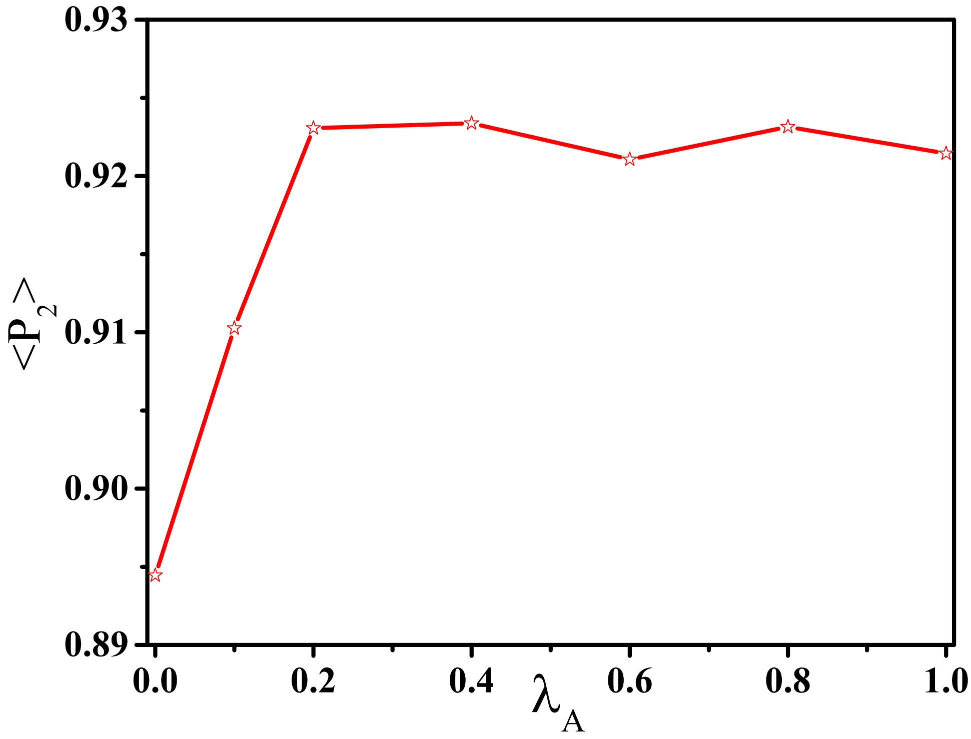

Fig. S9 The orientation degree $< P_2 >$ of the nanorods (NR) with respect to the NR functionalization degree (λ_A). (T^*=1.0, φ=4.0%, $\dot{\gamma}$=0.1)

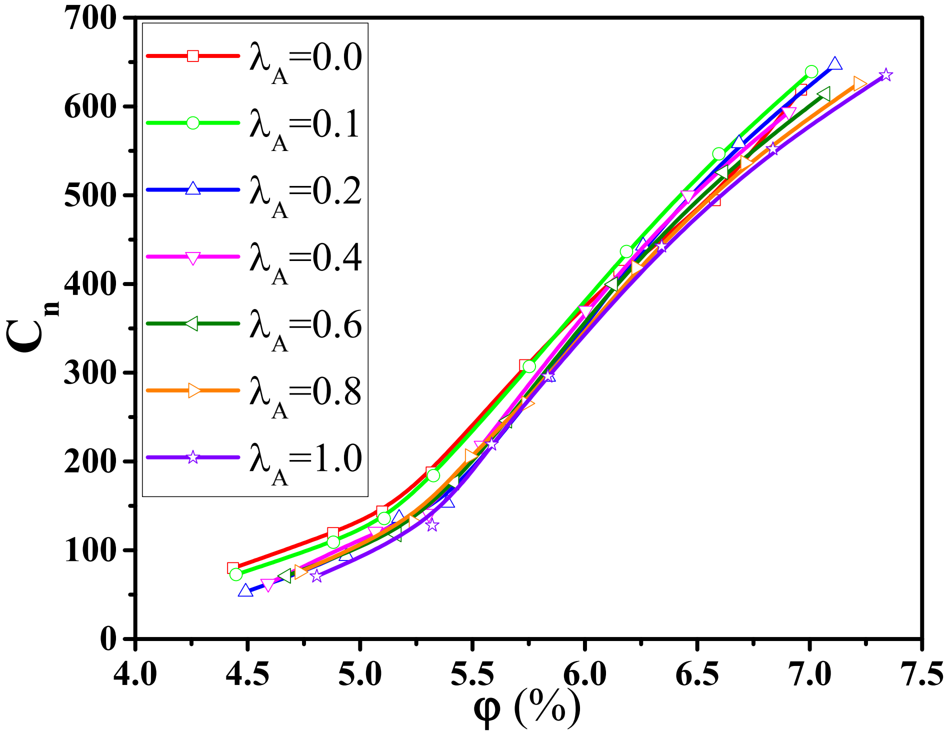

Fig. S10 Change of the main cluster size C_n as a function of the nanorod (NR) volume fraction φ for different NR functionalization degree (λ_A). (T^* =1.0, $\dot{\gamma}$=0.1)

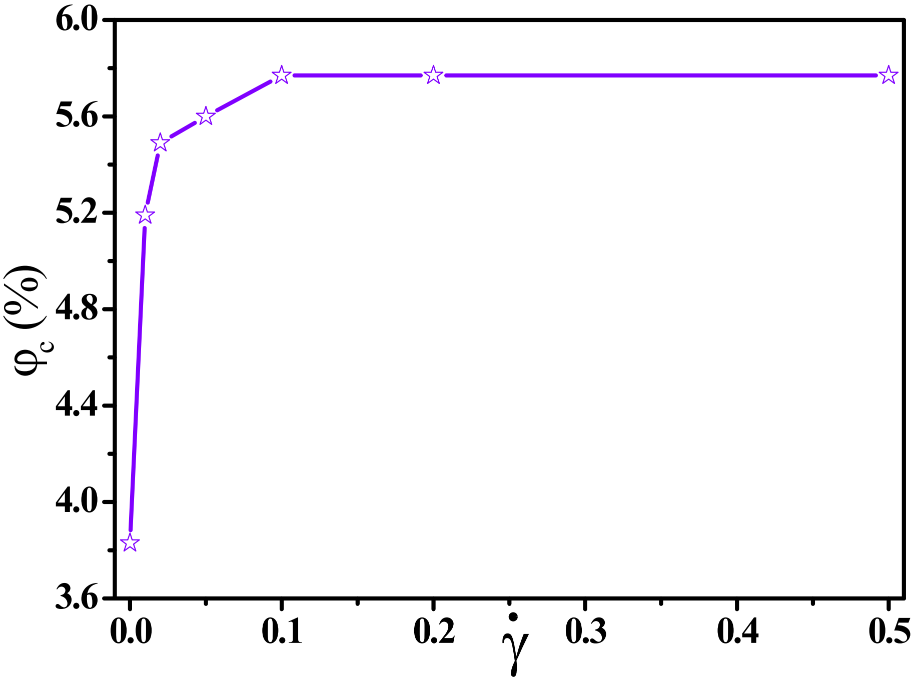

Fig. 11 The percolation threshold φ_c with respect to the shear rate $\dot{\gamma}$. (T^* =1.0, λ_A =0.1)

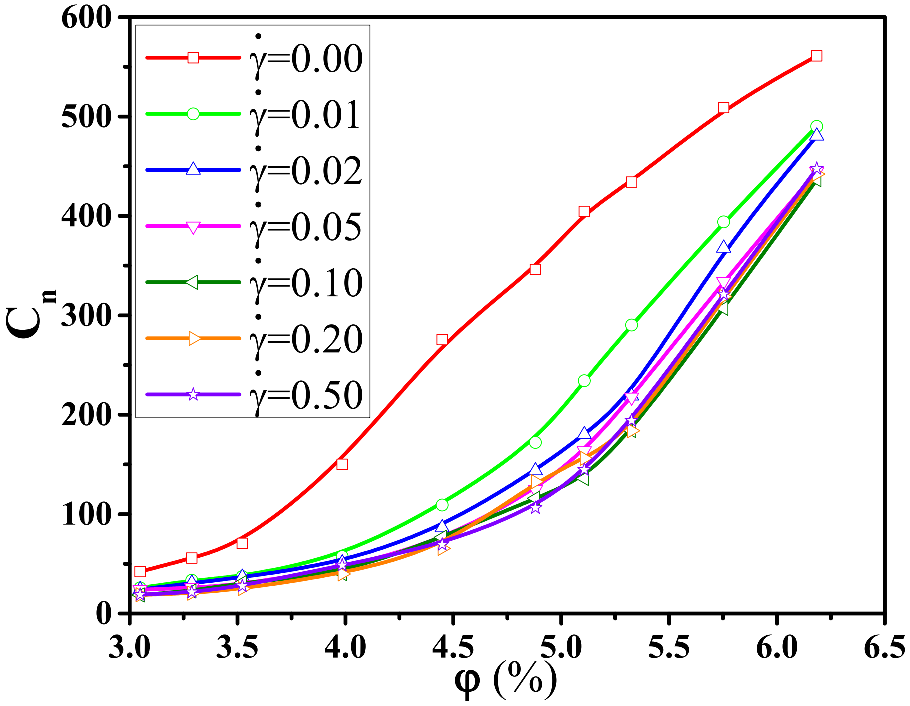

Fig. S12 Change of the main cluster size C_n as a function of the nanorod volume fraction φ for different shear rate ($\dot{\gamma}$). (T^* =1.0, λ_A =0.1)

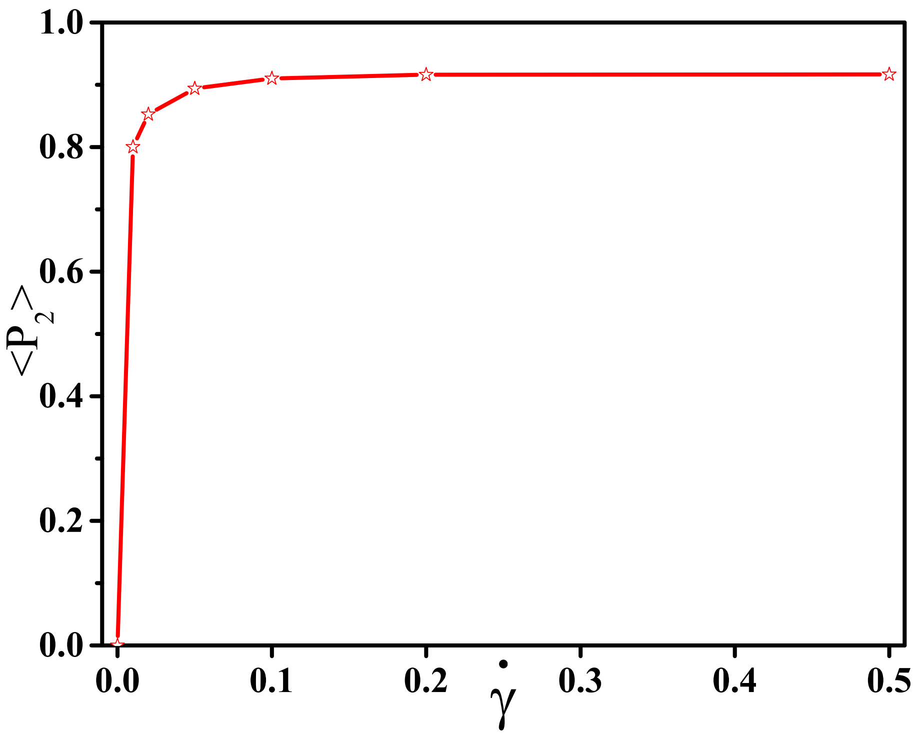

Fig. S13 the NR orientation $< P_2 >$ with respect to the shear rate $\dot{\gamma}$. (T^* =1.0, $\varphi = 4.0\%$, λ_A =0.1)

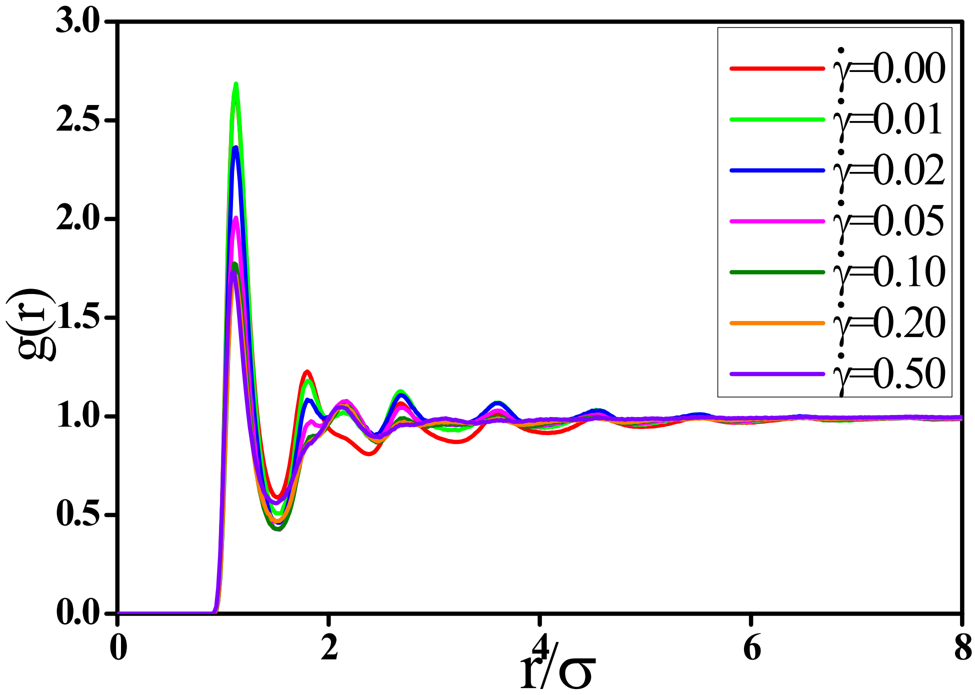

Fig. S14 RDF of nanorods with respect to the shear rate $\dot{\gamma}$. (T^* =1.0, $\varphi = 4.0\%$, λ_A =0.1)

Table S1 Nonbonded interaction parameters used in this work.

Interaction types	$\varepsilon_{ij}{}^{a}$ (ε)	$r_{cutoff}{}^{b}$ (σ)
PB^c-PB^c	1.0	$2\times 2^{1/6}$
PB^c-NR^u	1.0	2.5
PB^c-NR^f	1.0-5.0	2.5
NR^u-NR^u	1.0	2.5
NR^u–NR^f	1.0	2.5
NR^f–NR^f	1.0	2.5

[a] ε_{ij} the energy parameters between interacting sites i and j.

[b] r_{cutoff} is the cut-off distance.

[c]PB is the bead on polymer chain.
[u]NR is the unfunctionalized bead on nanorod.
[f]NR is the functionalized bead on nanorod.
